# Sensing of EGTA Mediated Barrier Tissue Disruption with an Organic Transistor

**DOI:** 10.3390/bios3010044

**Published:** 2013-01-08

**Authors:** Scherrine Tria, Leslie H. Jimison, Adel Hama, Manuelle Bongo, Róisín M. Owens

**Affiliations:** Department of Bioelectronics, Ecole Nationale Supérieure des Mines, CMP-EMSE, MOC 880 Rue de Mimet, Gardanne 13541, France; E-Mails: stria@emse.fr (S.T.); jimison@emse.fr (L.H.J.); hama@emse.fr (A.H.); bongo@emse.fr (M.B.)

**Keywords:** organic bioelectronics, tight junctions, paracellular transport, EGTA, barrier tissue, toxicology, biosensing, organic electrochemical transistor

## Abstract

Barrier tissue protects the body against external factors by restricting the passage of molecules. The gastrointestinal epithelium is an example of barrier tissue with the primary purpose of allowing the passage of ions and nutrients, while restricting the passage of pathogens and toxins. It is well known that the loss of barrier function can be instigated by a decrease in extracellular calcium levels, leading to changes in protein conformation and an increase in paracellular transport. In this study, ethylene glycol-bis(beta-aminoethyl ether)-*N*,*N*,*N'*,*N'*-tetra acetic acid (EGTA), a calcium chelator, was used to disrupt the gastrointestinal epithelial barrier. The effect of EGTA on barrier tissue was monitored by a novel label-free method based on an organic electrochemical transistor (OECT) integrated with living cells and validated against conventional methods for measuring barrier tissue integrity. We demonstrate that the OECT can detect breaches in barrier tissue upon exposure to EGTA with the same sensitivity as existing methods but with increased temporal resolution. Due to the potential of low cost processing techniques and the flexibility in design associated with organic electronics, the OECT has great potential for high-throughput, disposable sensing and diagnostics.

## 1. Introduction

Understanding and controlling the barrier function of epithelial tissue is of great importance for pharmaceutical research and drug development, as well as having applications in diagnostics and fundamental research. Manipulating barrier function is primarily applicable in targeted drug delivery, which involves the specific transport of a molecule across the lumen to the underlying tissue. Developing methods to achieve such transport is of particular relevance for drug delivery across gastrointestinal tissue. In some cases, an agent is used to temporarily increase permeability of the cell layer and allow passage of a drug [1]. Further, drug delivery assays require adequate *in vitro* cell models to assess drug transport. *In vitro* models for barrier tissue are also necessary to assess the effects of toxins or pathogens, for the purpose of both diagnostics and the study of diseases. Regardless of application, the goal of *in vitro* models is to mimic *in vivo* barrier tissue behavior. 

Barrier tissue generally consists of tightly packed layers of epithelial cells. Individual cells are joined to one another by junctional proteins, which act as cell-cell seals [2]. In addition, the cells are anchored to underlying tissues. The anchoring provides an asymmetric architecture to the barrier, in which the apical side is exposed to the lumen, and the basal side is attached to the basal lamina [3,4]. This architecture provides selective transport across the barrier, which can be modulated to increase the passage of nutrients *via* transient opening of the apical junction [5,6]. The apical junction is composed of two distinct junctions; the tight junction (TJ), found closest to the apical side, and the adherens junction (AJ), found underneath the TJ [7]. These junctions comprise complexes of intracellular and transmembrane proteins. The major proteins involved in TJs are claudins [8], occludins [9] and ZO-1 [10,11], while AJs consist primarily of E-cadherin and catenin [12]. Beneath the apical junction are additional junctional complexes known as desmosomes, which contribute to cell integrity [13]. The integrity of junctional protein complexes, and hence the integrity of barrier tissue, is known to be affected by outside stimuli. In particular, the function of some proteins such as cadherins, are sensitive to the concentration of extracellular calcium. Cadherins, found in both the adherens junction and the desmosome contain multiple calcium binding domains [14]. When insufficient calcium is present, cadherins are not able to form homo or heterojunctions with adjacent cells [15]. As a consequence, the proteins are internalized, leading to an opening of the paracellular pathway. Other tight junction associated proteins that require the presence of calcium include G proteins, protein kinase C and calmodulin [16]. Hence, decreases in extracellular calcium concentration can lead to disassembly of TJs. In fact, a calcium switch assay is often used to study TJ reformation after removal and then replacement of calcium [16].

In this study, we use Caco-2 cells grown on permeable transwell filters. When cultured in this format, these cells are known to form polarized monolayers with an apical brush border, similar to that found in the human colon [17]. More specifically, differentiated monolayers of Caco-2 cells create a barrier similar to that observed *in vivo*. Confluent monolayers of Caco-2 cells are widely used by the pharmaceutical industry to evaluate the absorption of oral drugs: the fraction of drug absorption can be directly correlated with the apparent permeability of the drug molecule across the cell layer [18]. The dependence of barrier integrity of the Caco-2 cells on calcium concentration has been confirmed [19], with the internalization of TJ and AJ proteins occurring when calcium concentrations decrease to micromolar levels [20]. As discussed above, disruption of junctional protein complexes leads to opening of the intercellular junction. To mimic this effect *in vitro*, we exposed Caco-2 monolayers to EGTA (Ethylene glycol-bis(beta-aminoethyl ether)-*N*,*N*,*N'*,*N'*-tetra acetic acid), a specific calcium chelator. EGTA has been shown previously to have dramatic effects on paracellular permeability and transepithelial resistance (TER) [1,21]. In particular, E-cadherin was found delocalized from the cell periphery after treatment with EGTA [22,23]. To monitor the barrier integrity of epithelial tissue on exposure to EGTA, we use a recently introduced method based on an organic electrochemical transistor (OECT) [24,25]. We validate the OECT results with traditional characterization techniques, including immunofluorescence staining of junctional adhesion proteins, permeability assays and measurement of transepithelial resistance (TER).

## 2. Experimental Section

**Cell Culture.** Caco-2 cells from ATCC were seeded at a density of 5 × 10^4^ cells/insert (1.1 cm^2^). Cells were routinely maintained at 37 °C in a humidified atmosphere of 5% CO_2_, in DMEM (Advanced DMEM Reduced Serum Medium 1×, Invitrogen) with 2 mM Glutamine (Glutamax™-1, Invitrogen), 10% FBS (Fetal Bovine Serum, Invitrogen) and Pen-strep (5,000 (U/mL) Penicillin-5000 (µg/mL) Streptomycin, Invitrogen). For all experiments, Caco-2 cell layers were used after 3 weeks in culture, corresponding to a TER of 400–500 Ω·cm^2^ and a maximum apparent permeability of 1 × 10^−6^ cm·s^−1^, consistent with literature reports [26]. Cells were cultured on transwell filters with a 0.4 µm² pore size and area of 1.1 cm^2^. For OECT measurements only, 24 transwell filters were used. Cells were exposed various concentrations of EGTA, from a stock solution of 0.6 M EGTA in DI water, pH adjusted to 7.4 with 1 M Tris-HCl. EGTA was added to the basal side of the cell filter, without changing media. Controls confirmed that the act of addition/removal of basal solution alone did not disrupt the barrier tissue layer.

**Immunofluorescence.** After exposure to EGTA, Caco-2 cells grown on filters were fixed with 3–4% paraformaldehyde in PBS pH 7.4, for 15 min at room temperature. Permeabilization was performed using 0.25% Triton in PBS, for 10 min at room temperature and with a blocking step consisting of 1% BSA in PBST (0.05% Tween 20 in PBS), for 30 min at room temperature. Mouse monoclonal anti-E-cadherin and rabbit polyclonal anti-claudin-1 and anti occludin were used at 5 µg/mL (Invitrogen), in 1% BSA in PBST for 1 h at room temperature. Monolayers were then incubated for 1 h at room temperature with the secondary antibodies Alexa Fluor 488 goat anti-mouse IgG and Alexa Fluor 568 goat anti-rabbit (Molecular Probes). Finally, the cells were incubated for 5 min at room temperature with Fluoroshield with DAPI (Sigma Aldrich), mounted and examined with a fluorescent microscope.

**Permeability Assays.** After exposure to EGTA, a permeability marker (Lucifer Yellow) was added to the apical side of the monolayer and fluorescence was measured after 1 h incubation at 37 °C in a humidified CO_2_ incubator. The value of the apparent permeability (*P_app_*) was calculated according to the following relationship: *P_app_ =* ((*Flux × V_bas_*)*/t*) *×* (*1/Co × A*) and *Flux = 100 ×* (*LY_bas_ × V_bas_*)*/*(*LY_api_ × V_api_*), where *LY_bas_* and *LY_api_* are the concentration of Lucifer Yellow in the basal and apical sides of the hanging porous filter, respectively, *V_bas_* and *V_api_* are the volume in the basal and apical sides, respectively, *t* is the time of incubation, *C_o_* is the initial concentration of Lucifer Yellow (LY) on the apical side and *A* is the area of the filter. At least two samples were measured for each condition. 

**CellZscope Measurements.** The CellZscope (Nanoanalytics) measures the impedance of barrier-forming cell cultures grown on permeable membranes and provides the transepithelial electrical resistance as output. Impedance of cell layers grown on filters as previously described, were measured in complete DMEM. During EGTA exposure, TER values were measured continuously.

**OECT Fabrication.** The conducting polymer formulation consisted of PEDOT:PSS (Heraeus, Clevios PH 1000), supplemented with ethylene glycol (Sigma Aldrich, 0.25 mL for 1 mL PEDOT:PSS solution), dodecylbenzenesulfonic acid (DBSA, 0.5 µL/mL), and 3-glycidoxypropyltrimethoxysilane (GOPS) (10 mg/mL), the latter serving as a heat activated cross-linker to ensure film stability in aqueous solutions. Devices were fabricated on glass slides with channel dimensions defined using a parylene peel-off technique described previously [27,28]. In this technique, a parylene film is deposited on glass and subsequently patterned using conventional photolithography techniques. PEDOT:PSS is deposited on the glass/parylene pattern. When the patterned parylene is removed from the glass substrate *via* mechanical peeling, PEDOT:PSS is left on the glass in the negative spaces. This technique allows the patterning of sensitive materials, in that it avoids exposure of the active layer (here, the conducting polymer) to harsh developers, solvents, or other etchants. Following PEDOT:PSS deposition, devices were baked for 1 h at 140 °C in atmospheric conditions. A PDMS well was added to confine the electrolyte and define the channel area. The OECTs in this study have a channel width of 2 mm and a channel length of approximately 6 mm, resulting in a channel area of approximately 12 mm^2^. PEDOT:PSS lines extending from the well served as the source and drain contacts.

**OECT Measurements.** For OECT measurements, Ag/AgCl served as the gate electrode. All measurements were made using a Keithley 2612 Source Meter and customized Labview software. Cell media (as described above) was used as the electrolyte. Measurements were performed at ambient temperature, but controls were conducted to ensure that temperature effects do not dominate changes in OECT response within the time required for measurements. Measurement parameters were chosen to avoid exposing the cell layers to a voltage drop above 0.5 V, as high voltages have been shown to damage bilayer membranes [29]. OECT data were collected using the following parameters: *V_DS_* = −0.2 V, *V_GS_* = 0.3 V, *V_GS_* on time = 2 s, off time = 28 s. Data are shown in the form of a normalized response of the OECT. The magnitude of modulation of drain current on application of a gate voltage pulse (*ΔI_D_)* is divided by the baseline current *I_o_*. The resulting *ΔI_D_/I_o_* dataset (unitless) is normalized to a [0,1] scale for easier data visualization and device to device comparison. Included in each measurement, but not shown, is a baseline of *ΔI_D_/I_o_* after cell layer removal, to aid in the assignment of NR = 1. In this way, NR = 1 refers to no barrier properties, and NR = 0 refers to full barrier properties of the cell layer (although not full suppression of OECT modulation [24]). See Appendix for further description.

## 3. Results and Discussion

**Figure 1:**
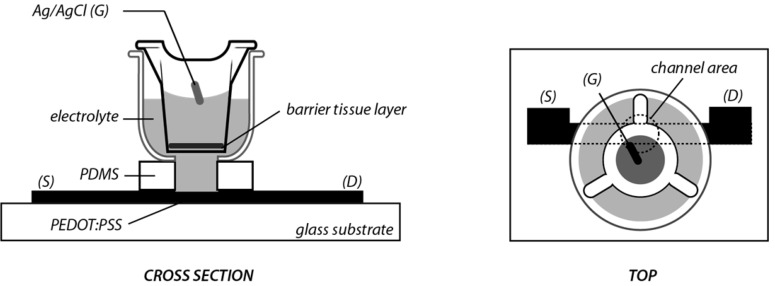
Illustration of organic electrochemical transistor (OECT) barrier tissue sensor. S, D, and G refer to the source, drain and gate electrodes. The channel area refers to the portion of PEDOT:PSS that is in contact with the electrolyte, and is defined by the PDMS well.

Barrier tissue cells grown on 24 well filters were integrated with an OECT as illustrated in Figure 1. A transwell filter hosting the cell layer is placed within an electrolyte well, between the Ag/AgCl gate electrode and the PEDOT:PSS channel.

### 3.1. OECT Measurement of EGTA Mediated Barrier Tissue Disruption

**Figure 2:**
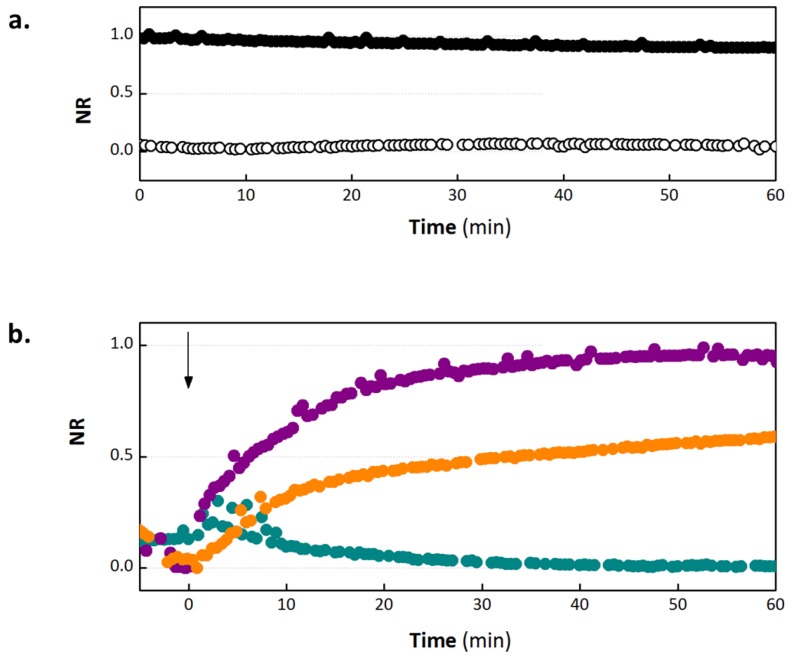
Normalized response of the OECT. (**a**) Control NR of OECT with cells (white) and without cells (black), with no exposure to ethylene glycol-bis(beta-aminoethyl ether)-*N*,*N*,*N'*,*N'*-tetra acetic acid (EGTA). (**b**) *In situ* monitoring of NR on addition of 1 mM (dark cyan), 10 mM (orange) and 100 mM (violet) EGTA. EGTA is added at time = 0, as indicated by the arrow.

In an OECT, the drain current (*I_D_*) between the source and drain electrodes is modulated by the application of a gate voltage. The mechanism for current modulation relies on the electrochemical doping and dedoping of a degenerately doped conducting polymer film in contact with an electrolyte [30,31]. A positive gate voltage induces a flux of positive ions into the transistor channel, dedoping the polymer film and reducing conductivity. On release of this potential, the ions leave the film and the original doping level, and hence conductivity, is restored. The *I_D_* transient response to a square gate voltage pulse is directly correlated with the magnitude of ionic flux into the conducting polymer. In the present device architecture, the barrier properties of the cell layer modify this ionic flux. Thus, monitoring the *I_D_* response to square *V_G_* pulses, shown in Figure 2 as normalized response (NR), yields information about the barrier properties of the cells with respect to ions in the electrolyte. Figure 2(a) shows the OECT normalized response as a function of time in the presence and absence of a cell layer. As illustrated in Figure 2(a), operation of the OECT with no cell layer corresponds to NR = 1, which is associated with a high ionic flux through the electrolyte and into the polymer channel. When intact barrier tissue is present, the ionic flux is reduced, and NR = 0. Figure 2(b) shows the OECT response in the presence of barrier tissue on addition of varying concentrations of EGTA. On introduction of 1 mM EGTA to the basolateral side of the transwell filter, we observe negligible changes in the normalized response of the OECT, indicating little or no disruption of barrier properties. There is perhaps a slight, transient increase in TER, which returns to baseline levels after 15 min. It is possible that there is a very slight decrease in ion flux. This has been observed with other compounds at low concentrations and may be explained by a tightening of the ion permeable TJ pores due to subtoxic exposure of certain chemicals [32]. Increases in TER have also been observed with an increase in permeability upon overexpression of occludin, illustrating functional decoupling of these two parameters [33]. 

On addition of 10 and 100 mM EGTA, we observe a concentration dependent reduction in NR, indicating increasing barrier disruption for higher concentrations of EGTA, with the presence of 100 mM EGTA destroying barrier integrity within 45 min. These experiments were carried out for 60 min only due to concerns that extended exposure of the cells to room temperature conditions would induce a change in barrier tissue properties [34]. It should be noted that in all experiments carried out in this study, EGTA was added directly to the cell medium. We anticipate that the use of EGTA in complete DMEM, as opposed to a calcium free buffer, will somewhat mitigate the effect of EGTA. However, as changes in both media and ionic composition are known to affect TER, we wanted to ensure that we saw effects due to calcium chelation alone. As a consequence, it is not surprising that we measure no effect on addition of 1 mM EGTA, as the calcium concentration in complete DMEM is approximately 1–2 mM and EGTA chelates calcium at a ratio of 1:1. In the case of 10 mM EGTA, the signal appears to become saturated but at an NR of slightly greater than 0.5. It is possible that this signal would continue to rise slowly, however we have previously observed a similar situation with low concentrations of hydrogen peroxide [24], where the signal reaches an intermediate level and then does not increase. This could represent a partial opening of the TJ or an inhomogeneous chelation of the calcium across the barrier tissue layer. An important feature of the OECT is the time in which breaches in barrier tissue integrity are detected. In the case of 100 mM EGTA, a 50% increase in NR is observed within 5 min, and the NR plateaus at a value slightly less than 1.0. We believe that the OECT can detect barrier tissue damage more rapidly because it is sensitive to more subtle breaches in barrier tissue integrity. The improved sensing speed has been observed on addition of a variety of toxic compounds (manuscripts in preparation). Of note, while the device is more sensitive to subtle barrier damage, the sensitivity is expected to suffer at the other extreme, suggesting that the present OECT is a poor sensor for the formation of barrier tissue and gross cell death/detachment.

### 3.2. Validation of EGTA Effect Using Immunofluorescence Staining of Junctional Proteins

**Figure 3:**
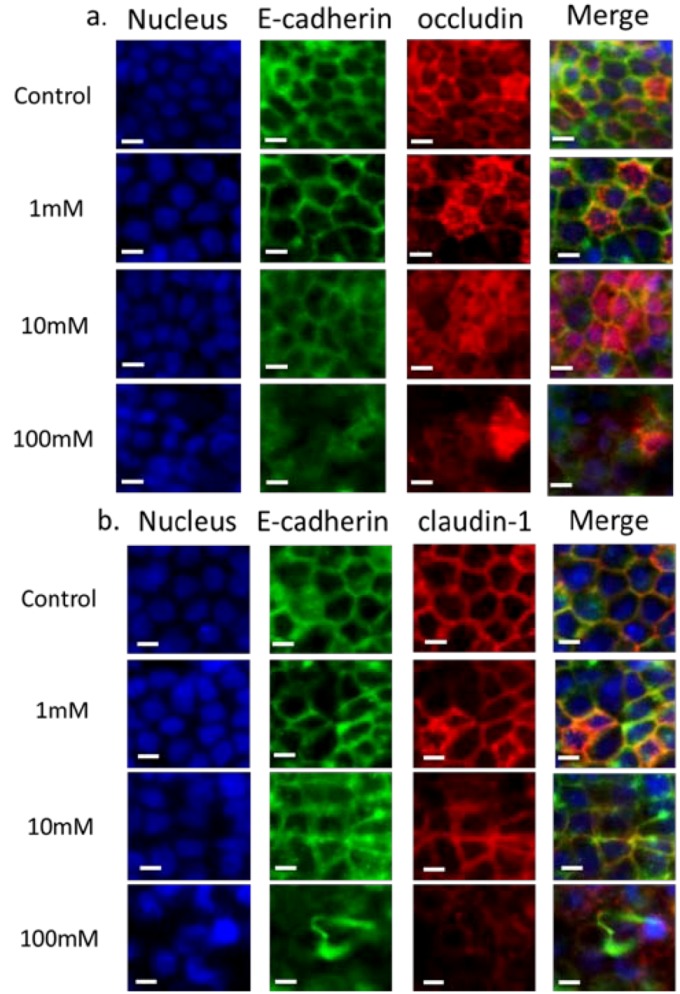
Immunofluorescence of proteins in the apical junction upon exposure to EGTA. Monolayers were exposed to various concentrations of EGTA for 2 h and then stained with antibodies against apical junction proteins. In panel **a**: DAPI (blue), E-cadherin (green) and occludin (red); in panel **b**: DAPI (blue), E-cadherin (green) and claudin-1 (red). Cells were exposed to 0, 1, 10 and 100 mM EGTA from top to bottom. The scale bar is 10 µm.

Figure 3 shows the immunofluorescence staining of adherens and tight junction proteins carried out after 2 h of exposure to EGTA using antibodies against E-cadherin, occludin and claudin-1. Cell nuclei were stained with DAPI. Control staining on Caco-2 cells without EGTA exposure shows that there is at least partial colocalization of E-cadherin with both occludin (Figure 3(a)) and claudin (Figure 3(b)), and that all three proteins are present at the cell peripheries. The colocalization of E-cadherin with occludin and claudin-1 is indicative of normal maturation and polarization of the cell layer as the presence of AJs are necessary to recruit TJs on the apical side to form apical junction complexes, resulting in an alignment of the AJs and TJs [35]. In the case of occludin the staining is slightly diffuse, the control included. This may partly be because immunofluorescence was carried out on filters rather than on coverslips, leading to slight warping of the substrate. On exposure to 1 mM EGTA, the proteins remain colocalized, with negligible difference compared to the control. On the addition of 10 mM EGTA, delocalization of proteins from the periphery is observed, indicating that the apical junctional complexes have been compromised. E-cadherin is considerably more diffuse in the sample exposed to 10 mM EGTA compared to the control. On exposure of 100 mM EGTA, E-cadherin, occludin and claudin-1 are present, but no longer localized at the cell borders. Previous studies have demonstrated that a 30 min treatment of 4 mM EGTA added to DMEM on the apical and basal side results in the displacement of occludin to the intracellular compartment [36], while a study which exposed Caco-2 cells to EGTA in media without calcium for 20 min [37] showed a normal localization of occludin with aberrant localization of claudin-1.

### 3.3. Validation of EGTA Effect Using CellZscope Measurement of TER and Permeability Assays

**Figure 4:**
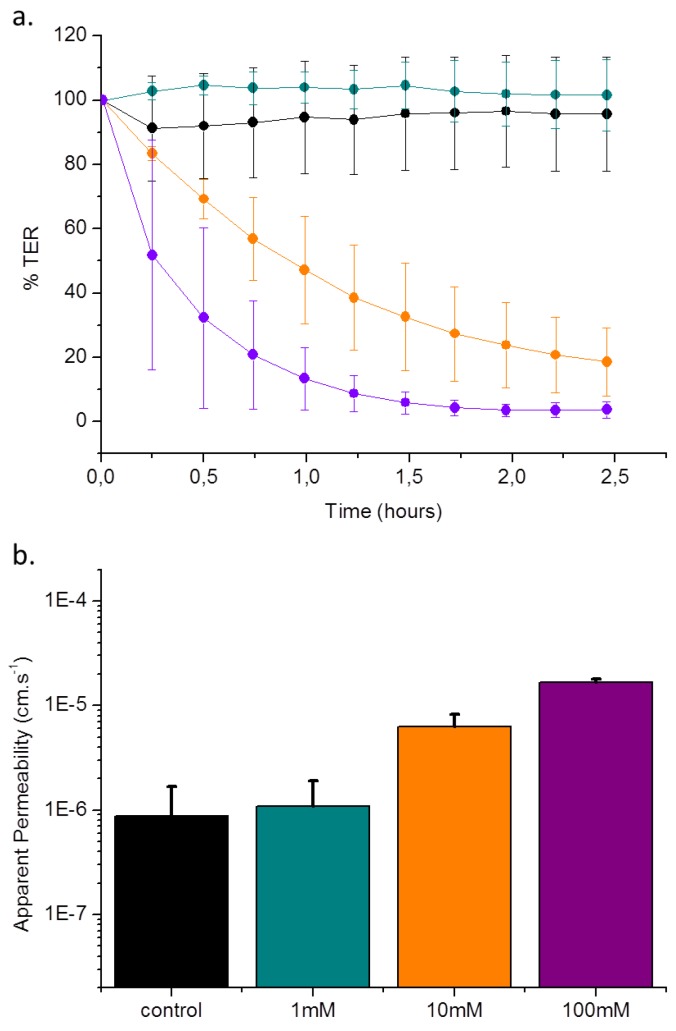
% TER value and permeability assays of cells upon exposure to EGTA. Monolayers were exposed to various concentrations of EGTA for 2.5 h. Panel **a** shows normalized TER value + standard deviation upon exposure to 0 mM (black), 1 mM (dark cyan), 10 mM (orange) and 100 mM EGTA (violet) for 2.5 h. During the experiment, measurements were taken continuously. Panel **b** shows the apparent permeability after exposure to EGTA. Data represents mean value + standard deviation of experiments made in duplicate.

Figure 4(a) shows the effect of EGTA on TER, as measured with the CellZScope. As seen with the OECT, exposure to 1 mM EGTA shows a slight change in TER with respect to the control. On exposure of 10 mM EGTA for two and half hours, the mean TER is reduced to approximately 20% of the initial value. On addition of 100 mM EGTA, the reduction in TER is faster, stabilizing at 10% of the initial value. To directly compare the rate of sensing with the OECT, a useful parameter to consider is the time taken to reach a 50% decrease in TER. As mentioned above, the OECT detects a 50% change (increase in NR) within approximately 5 min. In the case of the CellZscope, this change takes approximately 15 min. The time taken to reach a minimum TER value with 100 mM EGTA was 1.5–2 h, compared to 45 min with the OECT. It was previously demonstrated by the EVOM (epithelial Volt-Ohm meter) technique that in the presence of 1 mM EGTA on both sides of the monolayer, a rapid drop in TER value was observed in 10 min [38]. The discrepancy between these results and what we present here can be explained by the increased potency of EGTA when presented to both the apical and basal sides, further exaggerated by the use of a calcium free buffer [23]. 

Figure 4(b) shows results of permeability assays carried out on Caco-2 monolayers after 2 h of EGTA exposure. The addition of 1 mM EGTA leads to a permeability of (1.08 ± 0.81) × 10^−6^ cm·s^−1^, a slight increase compared to the control permeability of (8.81 ± 7.94) × 10^−7^ cm·s^−1^. The addition of 10 mM EGTA leads to an increase in permeability to (6.30 ± 1.95) × 10^−6^ cm·s^−1^, while treatment with 100 mM EGTA results in a permeability of (1.64 ± 0.14) × 10^−5^ cm·s^−1^, 18 times larger than the control value. For comparison, a filter alone typically has a permeability of 1 × 10^−4^ cm·s^−1^. Previous studies of Caco-2 permeability have demonstrated a mild increase on exposure of 2 mM EGTA in Krebs buffer, with a more marked increase at 20 mM EGTA (up to 4 hour exposure) [23]. 

Measurement of permeability, although a valuable parameter, must be considered carefully as the permeability measured is molecule-specific (depending on charge and size). Therefore, care must be taken when comparing these data with other parameters that describe barrier tissue integrity, such as TER. In general, a direct correlation between the solute permeability of a cell layer and the TER exist; tight cell layers exhibit high electrical resistance and low permeability, although there are certain conditions where these two parameters can be decoupled [39]. In fact, if the slight increase in permeability can be taken as statistically significant, it is in agreement with literature demonstrating that increases in TER can sometimes be accompanied by increases in permeability (discussed above), thus illustrating decoupling of these two parameters. This can be explained in part by the fact that TER is a parameter that describes an instantaneous snapshot of ion flux through claudin based pores in epithelial tissue layers [40], while permeability is a parameter that describes the transport of larger molecules, most likely through a different pathway, one theory being that larger molecules go through barrier tissue layers *via* dynamic opening and closing of tight junction strands. For this reason, permeability is a parameter that must be measured over longer time periods, e.g., 1 h. As indicated above, the permeability of Lucifer Yellow was measured after incubation of this compound for 1 hour (after 2 h exposure to EGTA). Therefore, for a fairer comparison, the latter time points should be considered for TER measurements. In the case of the results shown here, a clear increase in permeability is observed with 10 mM and 100 mM EGTA, in line with the trends observed with the OECT and the CellZscope.

## 4. Conclusions

In this study, we integrate cells with OECTs and demonstrate that the presence of a cell layer modulates the OECT transient response. Upon a decrease of extracellular calcium concentration due to the presence of EGTA, the OECT rapidly senses the breach in the epithelial cell layer in a concentration dependent manner. We validated these results by comparison with traditional techniques such as immunofluorescence, TER measurement and a Lucifer Yellow permeability assay, and found that the data correlates with the results obtained using the OECT. The OECT was able to detect EGTA-induced breaches in epithelial layers with equal sensitivity to the three techniques tested. Of the traditional techniques, only TER measurements (CellZScope) allow dynamic monitoring of barrier tissue integrity. Importantly, the OECT was found to be able to detect EGTA-induced breaches in epithelial layers with increased temporal resolution compared to the CellZscope. The OECT appears to be uniquely sensitive to subtle breaches in barrier tissue integrity, and measures with an extremely fine temporal resolution. We previously showed that the OECT was capable of measuring disruption of barrier tissue caused by exposure to hydrogen peroxide with 30 s [24]. Currently, the device probes the barrier tissue for a duration of 2 s, followed by a 28 off time, to realize a 30 s temporal resolution. The off time of the device serves two purposes: to give the cells adequate time to recover from the voltage pulse, and to allow for the recovery of the PEDOT:PSS film, bringing the drain current back to a baseline value. Duty cycle, and pulse duration could be optimized to observe more dynamic behavior if desired, with the limiting parameter being recovery of baseline current. For sake of comparison, the CellZscope requires 40 s to collect an impedance scan, which puts a hard limit on its temporal resolution. 

The CellZscope has an additional advantage in that it has been developed for use in an incubator, allowing continuous, real time monitoring of barrier tissue properties. Although the current prototype of the OECT allows dynamic measurement, it is limited to operation at room temperature. Work is ongoing to transition the OECT to an *in situ* measurement system to allow integration of traditional cell culture materials and electronics into an incubator to allow longer term measurements and further to streamline the operation for medium to high throughput testing. Future optimization of the OECT device for sensitivity is ongoing;the device is also being adapted for use with additional cell lines to create new tools in different diagnostic applications. In addition, we intend to capitalize on the advantages in low cost fabrication and ease of device design facilitated by the use of conducting polymers towards the goal of high throughput, disposable sensing and diagnostics. 

## Figures and Tables

**Figure S1:**
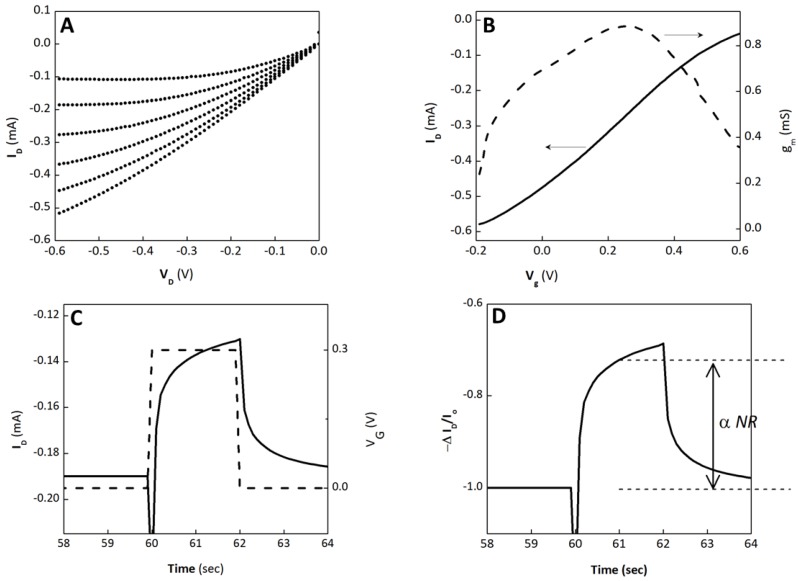
OECT Characteristics (no cells present.) (**A**) Output characteristics of the OECT used for barrier tissue sensing. (**B**) Transfer characteristics (solid line) and transconductance (dashed) for V_D_ = −0.6 V. The transconductance peaks near V_G_ = 0.3 V, the operating V_G_ of the OECT in sensing measurements. (**C**) I_D_ transient response (solid) to a square V_G_ pulse (dashed). (**D**) Normalized modulation, ΔI_D_/I_o_, for the same V_G_ pulse shown in (C). The dashed lines indicate the value extracted from data. Extracted values are subsequently normalized to a [0,1] dataset to obtain normalized response (NR). ΔI_D_/I_o_ values collected prior to sensing (no cells present) are used to define the upper extreme (NR = 1), while ΔI_D_/I_o_ values collected immediately after cells are introduced is used to define the lower extreme (NR = 0).

## References

[1] Boulenc X., Marti E., Joyeux H., Roques C., Berger Y., Fabre G. (1993). Importance of the paracellular pathway for the transport of a new bisphosphonate using the human Caco-2 monolayers model. Biochem. Pharmacol..

[2] Farquhar M.G., Palade G.E. (1963). Junctional complexes in various epithelia. J. Cell Biol..

[3] Gaillard J.L., Finlay B.B. (1996). Effect of cell polarization and differentiation on entry of listeria monocytogenes into the enterocyte-like Caco-2 cell line. Infec. Immunity.

[4] Anderson J.M., Balda M.S., Fanning A.S. (1993). The structure and regulation of tight junctions. Curr. Opin. Cell Biol..

[5] Anderson J.M. (2001). Molecular structure of tight junctions and their role in epithelial transport. News Physiol. Sci..

[6] Anderson J.M., van Itallie C.M. (1999). Tight junctions: Closing in on the seal. Curr. Biol..

[7] Guttman J.A., Finlay B.B. (2009). Tight junctions as targets of infectious agents. Biochim. Biophys. Acta.

[8] Colegio O.R., van Itallie C.M., McCrea H.J., Rahner C., Anderson J.M. (2002). Claudins create charge-selective channels in the paracellular pathway between epithelial cells. Am. J. Physiol. Cell Physiol..

[9] Matter K., Balda M.S. (1999). Occludin and the functions of tight junctions. Int. Rev. Cytol..

[10] Anderson J.M., Stevenson B.R., Goodenough D.A., Mooseker M.S. (1986). Molecular characterization of zo-1, a peripheral membrane-protein of the tight junction. J. Cell Biol..

[11] Fanning A.S., van Itallie C.M., Anderson J.M. (2012). Zonula occludens-1 and-2 regulate apical cell structure and the zonula adherens cytoskeleton in polarized epithelia. Mol. Biol. Cell.

[12] Baum B., Georgiou M. (2011). Dynamics of adherens junctions in epithelial establishment, maintenance, and remodeling. J. Cell Biol..

[13] Kowalczyk A.P., Bornslaeger E.A., Norvell S.M., Palka H.L., Green K.J. (1999). Desmosomes: Intercellular adhesive junctions specialized for attachment of intermediate filaments. Int. Rev. Cytol..

[14] Angst B.D., Marcozzi C., Magee A.I. (2001). The cadherin superfamily: Diversity in form and function. J. Cell Sci..

[15] Nagar B., Overduin M., Ikura M., Rini J.M. (1996). Structural basis of calcium-induced e-cadherin rigidification and dimerization. Nature.

[16] Balda M.S., Gonzalez-Mariscal L., Contreras R.G., Macias-Silva M., Torres-Marquez M.E., Garcia-Sainz J.A., Cereijido M. (1991). Assembly and sealing of tight junctions: Possible participation of G-proteins, phospholipase C, protein kinase C and calmodulin. J. Membrane Biol..

[17] Sambuy Y., De Angelis I., Ranaldi G., Scarino M.L., Stammati A., Zucco F. (2005). The Caco-2 cell line as a model of the intestinal barrier: Influence of cell and culture-related factors on Caco-2 cell functional characteristics. Cell Biol. Toxicol..

[18] Artursson P., Karlsson J. (1991). Correlation between oral drug absorption in humans and apparent drug permeability coefficients in human intestinal epithelial (Caco-2) cells. Biochem. Biophys. Res. Commun..

[19] Artursson P., Magnusson C. (1990). Epithelial transport of drugs in cell culture. II: Effect of extracellular calcium concentration on the paracellular transport of drugs of different lipophilicities across monolayers of intestinal epithelial (Caco-2) cells. J. Pharm. Sci..

[20] Ivanov A.I., Nusrat A., Parkos C.A. (2004). Endocytosis of epithelial apical junctional proteins by a clathrin-mediated pathway into a unique storage compartment. Mol. Biol. Cell.

[21] Artursson P. (1990). Epithelial transport of drugs in cell culture. I: A model for studying the passive diffusion of drugs over intestinal absorptive (Caco-2) cells. J. Pharm. Sci..

[22] Raiman J., Tormalehto S., Yritys K., Junginger H.E., Monkkonen J. (2003). Effects of various absorption enhancers on transport of clodronate through Caco-2 cells. Int. J. Pharm..

[23] Collares-Buzato C.B., McEwan G.T., Jepson M.A., Simmons N.L., Hirst B.H. (1994). Paracellular barrier and junctional protein distribution depend on basolateral extracellular Ca^2+^ in cultured epithelia. Biochim. Biophys. Acta.

[24] Jimison L.H., Tria S.A., Khodagholy D., Gurfinkel M., Lanzarini E., Hama A., Malliaras G.G., Owens R.M. (2012). Measurement of barrier tissue integrity with an organic electrochemical transistor. Adv. Mater..

[25] Tria S.A., Jimison L.H., Hama A., Bongo M., Owens R.M. (2012). Validation of the organic electrochemical transistor for *in vitro* toxicology. BBA-Gen. Subjects.

[26] Weber C.R., Shen L., Wu L., Wang Y., Turner J.R. (2011). Occludin is required for tumor necrosis factor (TNF)-mediated regulation of tight junction (TJ) barrier function. Gastroenterology.

[27] DeFranco J.A., Schmidt B.S., Lipson M., Malliaras G.G. (2006). Photolithographic patterning of organic electronic materials. Org. Electron..

[28] Khodagholy D., Gurfinkel M., Stavrinidou E., Leleux P., Herve T., Sanaur S., Malliaras G.G. (2011). High speed and high density organic electrochemical transistor arrays. Appl. Phys. Lett..

[29] Bernards D.A., Malliaras G.G., Toombes G.E.S., Gruner S.M. (2006). Gating of an organic transistor through a bilayer lipid membrane with ion channels. Appl. Phys. Lett..

[30] Bernards D.A., Malliaras G.G. (2007). Steady-state and transient behavior of organic electrochemical transistors. Adv. Funct. Mater..

[31] White H.S., Kittlesen G.P., Wrighton M.S. (1984). Chemical derivatization of an array of three gold microelectrodes with polypyrrole: Fabrication of a molecule-based transistor. J. Am. Chem. Soc..

[32] Moyes S.M., Morris J.F., Carr K.E. (2011). Roles of pre-treatment time and junctional proteins in Caco-2 cell microparticle uptake. Int. J. Pharm..

[33] Balda M.S., Whitney J.A., Flores C., Gonzalez S., Cereijido M., Matter K. (1996). Functional dissociation of paracellular permeability and transepithelial electrical resistance and disruption of the apical-basolateral intramembrane diffusion barrier by expression of a mutant tight junction membrane protein. J. Cell Biol..

[34] Armitage W.J., Juss B.K., Easty D.L. (1994). Response of epithelial (mdck) cell junctions to calcium removal and osmotic stress is influenced by temperature. Cryobiology.

[35] Miyoshi J., Takai Y. (2005). Molecular perspective on tight-junction assembly and epithelial polarity. Adv. Drug Deliv. Rev..

[36] Sheth P., Samak G., Shull J.A., Seth A., Rao R. (2009). Protein phosphatase 2A plays a role in hydrogen peroxide-induced disruption of tight junctions in Caco-2 cell monolayers. Biochem. J..

[37] Rothen-Rutishauser B., Riesen F.K., Braun A., Gunthert M., Wunderli-Allenspach H. (2002). Dynamics of tight and adherens junctions under egta treatment. J. Membrane Biol..

[38] Ma T.Y., Tran D., Hoa N., Nguyen D., Merryfield M., Tarnawski A. (2000). Mechanism of extracellular calcium regulation of intestinal epithelial tight junction permeability: Role of cytoskeletal involvement. Microsc. Res. Technique.

[39] Balda M.S., Whitney J.A., Flores C., Gonzalez S., Cereijido M., Matter K. (1996). Functional dissociation of paracellular permeability and transepithelial electrical resistance and disruption of the apical-basolateral intramembrane diffusion barrier by expression of a mutant tight junction membrane protein. J. Cell Biol..

[40] Van Itallie C.M., Fanning A.S., Bridges A., Anderson J.M. (2009). Zo-1 stabilizes the tight junction solute barrier through coupling to the perijunctional cytoskeleton. Mol. Biol. Cell.

